# Freshwater Colonization, Adaptation, and Genomic Divergence in Threespine Stickleback

**DOI:** 10.1093/icb/icac071

**Published:** 2022-06-06

**Authors:** Windsor E Aguirre, Kerry Reid, Jessica Rivera, David C Heins, Krishna R Veeramah, Michael A Bell

**Affiliations:** Department of Biological Sciences, DePaul University, Chicago, IL 60614, USA; School of Biological Sciences, Area of Ecology and Biodiversity, University of Hong Kong, Hong Kong, SAR 999077, China; Department of Ecology and Evolution, Stony Brook University, Stony Brook, NY 11794, USA; Institute of Evolutionary Biology and Environmental Studies, University of Zurich, Winterthurerstrasse 190, 8057 Zurich, Switzerland; Department of Ecology and Evolutionary Biology, Tulane University, New Orleans, LA 70118, USA; Department of Ecology and Evolution, Stony Brook University, Stony Brook, NY 11794, USA; University of California Museum of Paleontology, University of California, Berkeley, CA 94720, USA

## Abstract

The Threespine Stickleback is ancestrally a marine fish, but many marine populations breed in fresh water (i.e., are anadromous), facilitating their colonization of isolated freshwater habitats a few years after they form. Repeated adaptation to fresh water during at least 10 My and continuing today has led to Threespine Stickleback becoming a premier system to study rapid adaptation. Anadromous and freshwater stickleback breed in sympatry and may hybridize, resulting in introgression of freshwater-adaptive alleles into anadromous populations, where they are maintained at low frequencies as ancient standing genetic variation. Anadromous stickleback have accumulated hundreds of freshwater-adaptive alleles that are disbursed as few loci per marine individual and provide the basis for adaptation when they colonize fresh water. Recent whole-lake experiments in lakes around Cook Inlet, Alaska have revealed how astonishingly rapid and repeatable this process is, with the frequency of 40% of the identified freshwater-adaptive alleles increasing from negligible (∼1%) in the marine founder to ≥50% within ten generations in fresh water, and freshwater phenotypes evolving accordingly. These high rates of genomic and phenotypic evolution imply very intense directional selection on phenotypes of heterozygotes. Sexual recombination rapidly assembles freshwater-adaptive alleles that originated in different founders into multilocus freshwater haplotypes, and regions important for adaptation to freshwater have suppressed recombination that keeps advantageous alleles linked within large haploblocks. These large haploblocks are also older and appear to have accumulated linked advantageous mutations. The contemporary evolution of Threespine Stickleback has provided broadly applicable insights into the mechanisms that facilitate rapid adaptation.

## Introduction

Like many of his contemporaries, Charles Darwin (1859) inferred that the distribution of species in geological time and geographical space provide strong evidence for evolution as a product of biological history. Although common bent grass (*Agrostis capillaris*), which he had studied (Darwin Correspondence Project, accessed January 23, 2022), had evolved resistance to toxic soil on Roman copper mines in England (Darwin 1859; McNeilly 1968) asserted that evolution was too slow to detect in the present. Instead, he argued for natural selection by analogy with formation of domesticated breeds by means of artificial selection. Consequently, when Bateson (1900) reported industrial melanism (Kettlewell 1973; Brakefield and Liebert 2000; Majerus 2009; Cook and Saccheri 2013) and other cases of ongoing evolution came to light, they were regarded as unrepresentative outcomes of human habitat disturbance. One hundred and forty years later, Hendry and Kinnison (1999) focused attention on “contemporary evolution.” Many cases have been recognized since then, and they stimulated further research on contemporary evolution (Hendry et al. 2008).

**Fig. 1:**
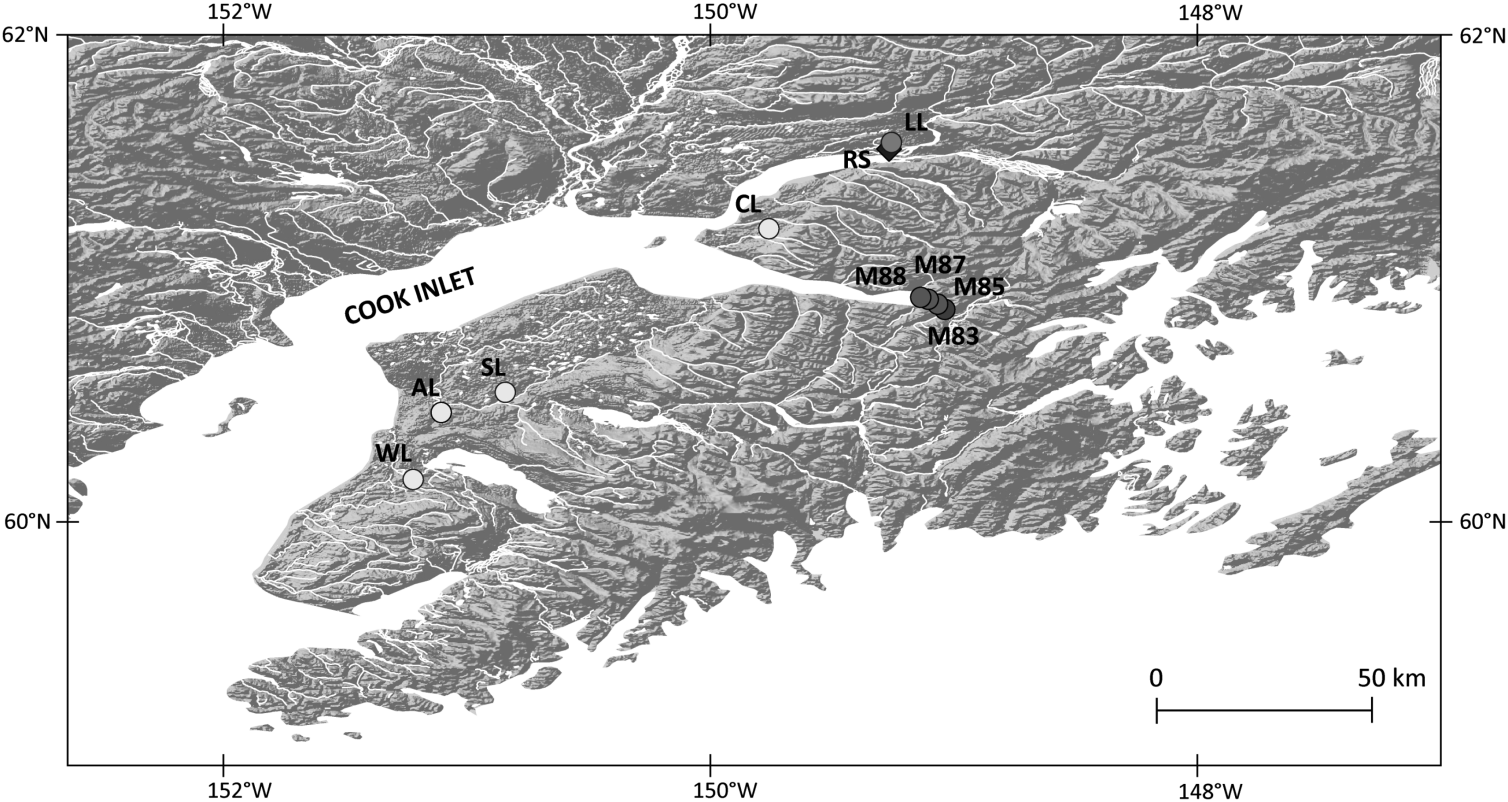
Locations of young freshwater populations (YFPs; circles) and the anadromous founder (i.e., RS, diamond) of three experimentally founded populations (i.e., CL, SL, WL) and the probable founder of one (i.e., LL) YFPs are represented by circles. Older populations are darker. See Table 1 for site acronyms and information on the populations.

**Fig. 2:**
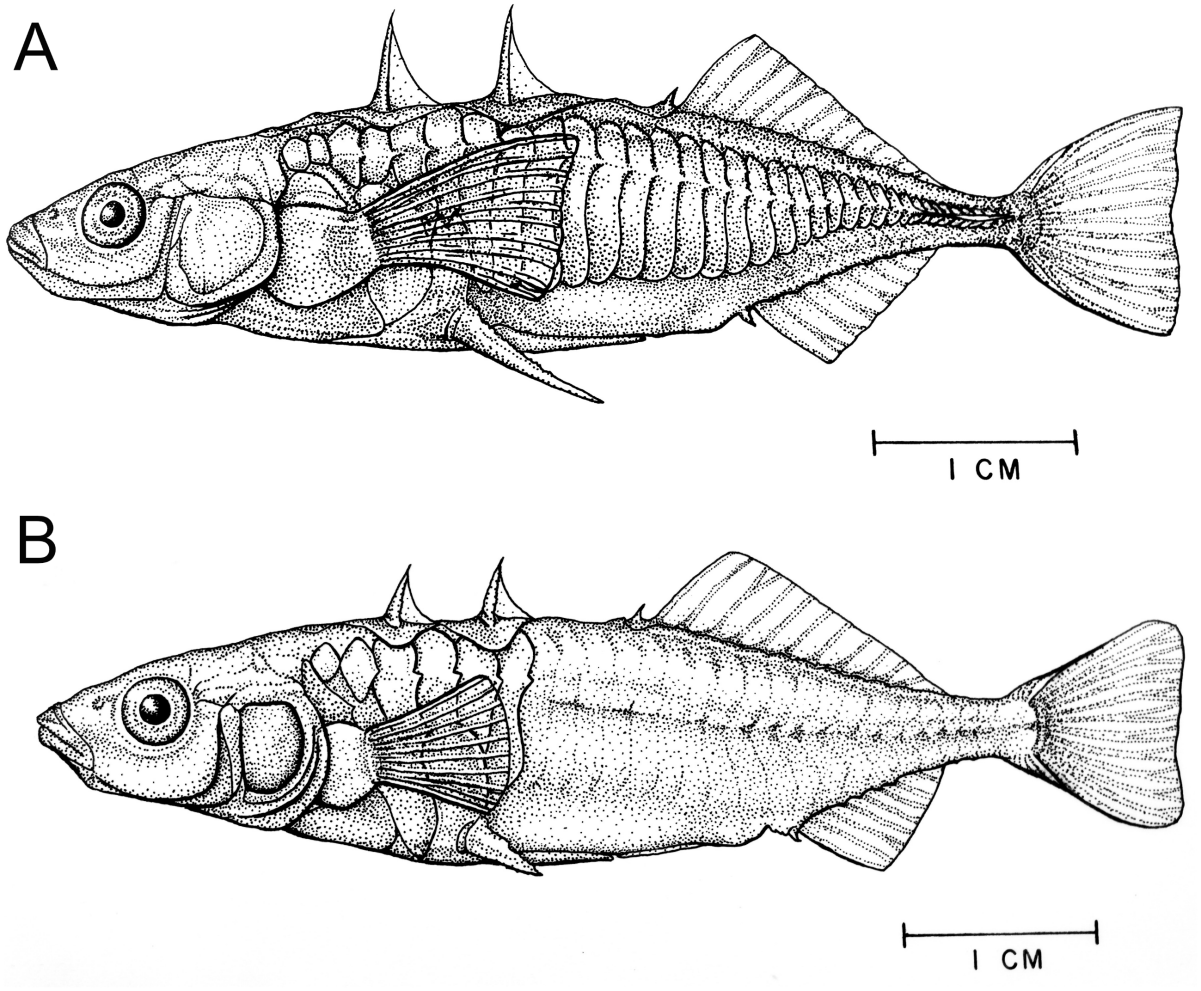
Completely plated anadromous (**A**) and low-plated freshwater (**B**) Threespine Stickleback. Note differences in armor plating, body shape, the sizes of the head, fins, and pelvic skeleton, and the position of the pectoral fin (Bell 1976).

By 1999, however, research on contemporary evolution in the Threespine Stickleback (*Gasterosteus aculeatus*) had already been published (Klepaker 1993; Hagen and Gilbertson 1973a), and we (the authors) had started to make annual samples from a stickleback population in Loberg Lake, Alaska (Bell 2001). This population apparently was founded naturally by anadromous stickleback (Fig. 1) after the native population was exterminated, and it had already begun to diverge for several phenotypic traits (Bell 2001; Bell et al. 2004; Arif et al. 2009; Aguirre and Bell 2012; Furin et al. 2012;Bell and Aguirre 2013) toward a freshwater phenotype (Fig. 2). Encouraged by the high rate of evolution and the large number of traits affected in the Loberg Lake stickleback population and by experimental results elsewhere (Barrett et al. 2008), we founded three experimental populations of Threespine Stickleback in three lakes around Cook Inlet, Alaska in 2009 (Cheney Lake), 2011 (Scout Lake), and 2019 (Warfle Lake) using about 3000, sexually mature, anadromous (i.e., sea-run) founders from Rabbit Slough per lake (Bell et al. 2016). Roberts Kingman et al. (2021) reported on genomic evolution in the Loberg, Cheney, and Scout lake populations, in which ∼40% (138/344) of the freshwater-adaptive allele frequencies changed from about 0 to 50% within just 8 y (i.e., approximately 5 generations) after founding as a result of positive selection. In this paper, we review our previous studies of contemporary phenotypic, genetic, and genomic evolution in Loberg, Cheney, and Scout lakes during the last several decades and comment briefly on six other Cook Inlet lake populations that were founded within the last 100 y by anadromous stickleback (Table 1).

**Table 1:** Lakes with young Threespine Stickleback populations in the Cook Inlet basin, Alaska

Site name	Latitude (degrees)	Longitude (degrees)	Code	Cause of habitat vacancy	Founding year	Type of founding	No. of founders
Rabbit Slough	61.532	–149.266	RS	NA	NA	NA	NA
Knik Lake	61.461	–149.733	KL	Rotenone	1982	Natural	Unknown
Loberg Lake	61.558	–149.256	LL	Rotenone	1982	Natural	Unknown
Cheney Lake	61.204	–149.76	CL	Rotenone	2009	Experimental	2964
Scout Lake	60.532	–150.842	SL	Rotenone	2011	Experimental	3047
Warfle Lake	60.174	–151.221	WL	Northern pike	2019	Experimental	2899
Mile 83	60.873	–149.037	M83	Railroad construction	1914	Natural	Unknown
Mile 85	60.894	–149.066	M85	Highway construction	1966	Natural	Unknown
Mile 87	60.914	–149.105	M87	Highway construction	1966	Natural	Unknown
Mile 88	60.922	–149.136	M88	Highway construction	1966	Natural	Unknown
Arc Lake	60.449	–151.105	AL	Rotenone	2008	Unknown	Unknown

The codes are used in Fig. 1 to identify populations. Founding year is known for experimentally founded populations but is maximal for naturally founded ones (i.e., based on year lake formed or the native population exterminated).

Studying contemporary evolution in freshwater Threespine Stickleback populations that were founded within the last few decades (hereafter referred to as “young freshwater populations”) by oceanic ancestors (i.e., anadromous or marine; Bell and Foster 1994a) can provide novel evolutionary insights. Stickleback biology is very thoroughly studied (Wootton 1976, 1984; Ziuganov V. 1991; Paepke 1996; Östland-Nilsson et al. 2007; von Hippel 2010; Bell and Foster 1994b) because adaptation to freshwater conditions occurs within a few decades and is manifested by changes of numerous traits. Evolutionary processes have been studied extensively in Threespine Stickleback, and it has become an attractive model to study evolutionary genetics and genomics (Gibson 2005; Kingsley and Peichel 2007; Reid et al. 2021). Evolution in small freshwater isolates is so fast that new mutations rarely play a major role in adaptation, and thus it is important to recognize that the contemporary evolutionary genetics and genomics of these populations must depend almost exclusively on ample freshwater-adaptive, standing genetic variation at hundreds of loci in the oceanic ancestors (Colosimo et al. 2005; Schluter and Conte 2009; Bell and Aguirre 2013; Roberts Kingman et al. 2021).

### The Threespine Stickleback is ancestrally marine

The closest relatives to the sticklebacks (family Gasterosteidae) are marine (Kawahara et al. 2009), and many Threespine Stickleback populations are oceanic, indicating that the ancestral state for *G. aculeatus* is most likely marine. The common occurrence of freshwater stickleback on islands (e.g., Iceland, Middleton Island, Alaska) or in fjords (e.g., Cook Inlet, Alaska) has resulted from repeated postglacial colonization of freshwater habitats from the sea since deglaciation within the last 20 Ky (e.g., Lindsey 1962; McPhail and Lindsey 1970; Bell 1976; Schluter and Conte 2009; Bell and Foster 1994a). Since anadromous Threespine Stickleback breed and start life in fresh water, they are clearly preadapted (exapted, *sensu*Gould and Vrba 1982) to colonize it. Thus, studies of evolution in YFPs of Threespine Stickleback can provide realistic insights into the evolutionary process that actually produced numerous, phenotypically divergent, resident freshwater populations along extensive Holarctic coastlines of North America and Eurasia, and throughout low-lying interior regions of Europe (Bassham et al. 2018; Terekhanova et al. 2014; Roberts Kingman et al. 2021).

### The antiquity of freshwater colonization

The earliest known fossil oceanic *G. aculeatus* is 13 My old (Bell et al. 2009), and there are several 10 My old stickleback records from marine and freshwater deposits (Bell 1994, 2009). Bell and Frank (unpublished data) used the chronological distribution of fossil Threespine Stickleback to estimate that the *G. aculeatus* species complex diverged from Ninespine Stickleback (*Pungitius pungitius*) 21 My old. Articulated fossil stickleback from marine and freshwater deposits resemble extant populations from those habitats, so freshwater colonization by oceanic stickleback and subsequent adaptive radiations there must have been occurring for at least 10 My. Since the divergent skeletal traits of freshwater stickleback fossils are similar to those of extant populations (Bell 1994, 2009), and many of these traits are strongly genetically determined (Miller et al. 2014; Peichel and Marques 2017), the alleles for adaptation to fresh water must have been accumulating for millions of years. This inference from the fossil record now has extensive genomic support (Nelson and Cresko 2018; Varadharajan et al. 2019; Roberts Kingman et al. 2021).

### Allelic recycling

Threespine Stickleback experience sharply contrasting conditions in marine and freshwater habitats. For example, the ocean is saline and clear, lacks structural refuge, and has diverse, large-mouthed, predatory fishes but no predatory insects. Colonization of fresh water exposes oceanic Threespine Stickleback to many dramatic ecological differences and intense directional natural selection (Roberts Kingman et al. 2021; Schluter et al. 2021). In northeastern Pacific stickleback, all but two of 21 chromosomes had at least one significant peak of divergence between oceanic and YFPs, and at least seven chromosomes had large clusters of peaks (Jones et al. 2012; Roberts Kingman et al. 2021). Although freshwater stickleback populations are highly divergent from their oceanic ancestors and isolated from each other within numerous separate drainages throughout their Holarctic range, they are highly convergent for numerous phenotypic traits (Bell and Foster 1994a). Remarkably, convergent phenotypic evolution in freshwater stickleback depends on hundreds of alleles that are often more closely related to each other than to homologous alleles that predominate in their marine ancestors (Colosimo et al. 2005; Jones et al. 2012; Roberts Kingman et al. 2021). Many of these freshwater-adaptive alleles occur at low frequencies in their oceanic ancestors throughout the northern hemisphere, and especially in the northeastern Pacific (Colosimo et al. 2005; Jones et al. 2012; Roberts Kingman et al. 2021).

### The “transporter hypothesis”

The crew of the fictional starship *Enterprise* traveled between distant locations using a “transporter machine,” which disaggregated their atoms at the point of departure and reaggregated them at the destination. Schluter and Conte (2009) used the transporter of the starship *Enterprise* as a metaphor for disaggregation of freshwater-adaptive alleles that have been introduced to anadromous stickleback populations by introgressive hybridization with freshwater residents, and their increase in frequency and reaggregation by sexual recombination during adaptation of oceanic stickleback populations to freshwater habitats that they have colonized.

Anadromous stickleback often enter freshwater habitats to breed and hybridize with freshwater resident stickleback, enabling flow of old, freshwater-adaptive alleles into anadromous populations by introgressive hybridization (e.g., Hagen 1967; Jones et al. 2006; Karve et al. 2008). Thus, when oceanic stickleback colonize fresh water, they already possess standing genetic variation that is adaptive for fresh water at hundreds of loci at the population level, but with each individual carrying only a small fraction of the freshwater-adaptive alleles (i.e., they are disaggregated) (Barrett and Schluter 2007; Nelson and Cresko 2018; Roberts Kingman et al. 2021). Natural selection in fresh water can increase the frequencies of individual freshwater-adaptive alleles rapidly, and sexual recombination can reaggregate them within individuals within four to ten generations into long freshwater haploblocks (Schluter and Conte 2009; Roberts Kingman et al. 2021). Similarly, freshwater haploblocks can re-enter marine environments through hybridization, allowing them to be disaggregated again, and ultimately be reconstituted in a distant freshwater location.

For example, freshwater populations are often monomorphic for the “low lateral plate morph” (i.e., <10 anterior lateral armor plates, Fig. 2B), but the oceanic populations from which they evolved independently are often monomorphic for the complete morph (i.e., ∼33 lateral plates from head to tail; Fig. 2A; Bell 1981). Colosimo et al. (2004, 2005) showed that the *Ectodysplasin* (*Eda*) gene has a large effect on lateral plate morphs, and that the low-morph *Eda* allele occurs as a rare variant in oceanic populations across the entire circumpolar distribution (see also Bell et al. 2010). The genomic region containing *Eda* is actually highly pleiotropic and associated with variation for plate number, neuromast number and pattern, body shape, and schooling behavior, traits that differ between oceanic and freshwater stickleback (Albert et al. 2007; Mills et al. 2014; Greenwood et al. 2016; Peichel and Marques 2017; Archambeault et al. 2020). The phylogenetic tree by Colosimo et al. (2005) was based on random DNA sequences around the Threespine Stickleback genome and was congruent with geographical distances among populations of both oceanic and freshwater stickleback throughout their Holarctic range. However, their gene tree based on the *Eda* region in the same set of populations had two major branches: one for almost all low morphs in freshwater populations and another for complete morphs in oceanic populations (Colosimo et al. 2005). Closely, related low morph alleles shared by geographically distant freshwater isolates must have been present as standing genetic variation in the founding oceanic ancestors (Colosimo et al. 2005). Jones et al. (2012) generalized this observation from *Eda*; many SNP variants that predominate in freshwater populations are also monophyletic with respect to homologous alleles that predominate in their oceanic ancestors. Roberts Kingman et al. (2021) confirmed and extended this finding to additional loci and increased the number of recycled genomic regions detected by increasing the number of sampled genomes 10-fold. Reuse of standing genetic variation of freshwater-adaptive alleles enhances divergence after freshwater colonization in the northeast Pacific, where *G. aculeatus* is much older than in the Atlantic (Bell 1994, 2009) and contains freshwater-adaptive alleles approximately five times as many loci detected in north Atlantic populations (Fang et al. 2020; Magalhaes et al. 2021). A substantial number of these freshwater-adaptive alleles are more than 1 My old, substantially older than many current populations in which they are circulating.

### Theoretical utility and limits of recently founded freshwater Threespine Stickleback populations

YFPs offer exceptional opportunities to study the dynamics of phenotypic, allele frequency, and whole genome evolution. The rapid evolution of these populations enables analysis of evolutionary dynamics over a few generations and even between successive generations (Bell and Aguirre 2013; Lescak et al. 2015; Roberts Kingman et al. 2021; Schluter et al. 2021; Garcia‐Elfring et al. 2021). With a typical generation time of one or two years (Baker 1994), it is practical to use long evolutionary time series from YFPs to study evolutionary dynamics.

Many young freshwater stickleback populations have originated after extermination of native populations (Bell and Aguirre 2013; Bell et al. 2016), construction of impoundments (Klepaker 1993; Bell and von Hippel unpublished data), earthquakes (Gelmond et al. 2009; Lescak et al. 2015), or deglaciation (von Hippel and Weigner 2004) created vacant or new freshwater habitats that were accessible to Threespine Stickleback only from the ocean. Although geographically adjacent YFPs are likely to be derived from the same or genetically similar oceanic populations (Withler and McPhail 1985; Taylor and McPhail 1999, 2000), they each experience separate demographic histories and environmental conditions. Even though they are not true replicates, geographically adjacent, young, freshwater populations should have similar genetic variation and adapt to habitats that contrast consistently with the ancestral marine environment.

Evolutionary theory has focused primarily on the evolutionary response to selection on phenotypes determined by new mutant alleles (Fisher 1930; Orr 1998). New mutant alleles are random with respect to fitness, unlikely to increase it, and they appear as single copies that are likely to be lost by genetic drift, limiting their potential to respond quickly to new environments (Barrett and Schluter 2007). In contrast, multiple copies of freshwater-adaptive alleles will usually be present in founding oceanic populations, and are less likely to be lost by drift, making adaptation from such standing genetic variation a more plausible mechanism for repeated and rapid evolution (Hermisson 2005; Matuszewski et al. 2015; Bell et al. 2016).

Finally, it is possible that evolution observed in young freshwater stickleback populations is common but rarely observed in other species. For example, the classic case of industrial melanism in British peppered moths was noticed because many amateur lepidopterists in Great Britain had collected peppered moths (*Biston betularia*) in many places over many decades (Cook and Saccheri 2013). Contemporary evolution in Threespine Stickleback (Bell and Aguirre 2013), Darwin's finches (Grant and Grant 2002; Lamichhaney et al. 2017), and *Anolis* lizards (Stuart et al. 2014) were noticed in the course of other research. The results of research on contemporary evolution in YFPs of Threespine Stickleback may be broadly applicable.

### Young freshwater Threespine Stickleback populations in Cook Inlet lakes

In the Cook Inlet basin and adjacent Kenai Peninsula, Alaska, there is a total of 10 lake populations that we know or believe were founded by anadromous Threespine Stickleback within about the last 100 y (Fig. 1, Table 1). We founded populations in Cheney, Scout, and Warfle lakes with anadromous stickleback (Table 1) and have sampled them annually (Bell et al. 2016). The other putative YFPs were naturally founded. We have studied the Loberg Lake population more extensively than any of the others (Aguirre and Bell 2012; Roberts Kingman et al. 2021). Stickleback in Knik Lake were exterminated in 1957 and 1982, and it apparently was recolonized by anadromous stickleback that evolved unusual lateral plate phenotypes (Francis et al. 1985). Three impoundments near Girdwood, Alaska at miles 85, 87, and 88 of Seward Highway formed after the highway was reconstructed following the 1964 Great Alaska Earthquake. These impoundments must have been colonized by anadromous stickleback after the highway was repaired. An adjacent lake at mile 83 formed in about 1914, after construction of the Alaska Railroad grade. Freshwater stickleback in all four of these lakes breed in sympatry with anadromous stickleback from which they are phenotypically divergent (Bell and von Hippel, unpublished data). Arc Lake was treated with rotenone to exterminate fishes in 2008 (Massengill unpublished report). Threespine Stickleback sampled from it annually since 2018 closely resemble anadromous stickleback, which apparently colonized it since 2008 (Bell unpublished data). Contemporary evolution in these young populations is individually interesting, and comparisons among them and YFPs elsewhere (e.g., Klepaker 1993; Gelmond et al. 2009; Terekhanova et al. 2014; Lescak et al. 2015) can produce general insights into evolutionary processes.

### Contemporary phenotypic evolution in young lake populations founded recently by anadromous Threespine Stickleback

**Fig. 3:**
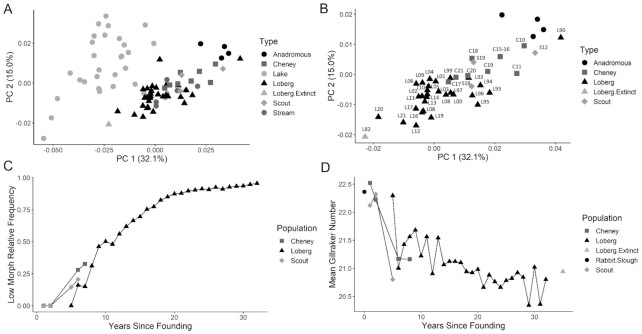
Contemporary phenotypic evolution in YFPs in Loberg (L), Cheney (C), and Scout (S) lakes. Points are sample means. The earliest values in each lake are similar to the anadromous Rabbit Slough ancestor and the latest approximate that of the original Loberg population. (**A**) Principal component analysis (PCA) of body shape variation in YFPs. Numbers in parentheses on the axes are the percentage of variation accounted for by each PC. Typical anadromous and established lake and stream populations from the Cook Inlet basin, Alaska, and time series from the three young populations included in the analysis. (**B**) Closeup of body-shape evolution in the time series as in (**A**) for the three young populations labeled with lake name and the last two digits of the collection year. (**C**) Evolution of the relative frequency of low plate morphs (see Fig. 2B) in the the three young lake populations. The x-axis indicates years since the populations were founded by anadromous stickleback. Values for Years Since Founding for Loberg Lake points are estimates. (**D**) Evolution of the mean gill-raker number in the three young lake populations. The x-axis is the same as in (**C**).

The extant Loberg Lake stickleback population was established through unknown means by anadromous stickleback after the lake was treated with rotenone in 1982 and has been sampled annually since 1990 (Bell 2001; Bell et al. 2004; Bell and Aguirre 2013). The Cheney and Scout lake populations were established in 2009 and 2011, respectively, using about 3000 anadromous stickleback from Rabbit Slough following their treatment with rotenone to exterminate invasive northern Pike, *Esox lucius* (Bell et al. 2016; Table 1). Pike predation in Warfle Lake (the most recently seeded lake) eliminated the stickleback population, and then pike were exterminated by exhaustive gill netting (Massengill pers. comm.). Phenotypes of the Loberg, Cheney, and Scout lake populations are evolving rapidly in the direction of established resident freshwater populations in the area for every morphological trait we have examined (Bell and Aguirre 2013), including body shape (Fig. 3A and B), lateral plate morphs (Fig. 3C), operculum shape (Arif et al. 2009), low morph plate number (Bell et al. 2004), and gill-raker number (Fig. 3D; Rivera, unpublished data). There is also evidence that their life history traits are evolving (Kurz et al. 2016; Baker et al. 2019). There has been consistent evolution of the frequencies of lateral plate morphs and the major gene for them (i.e., *Ectodysplasin*; Colosimo et al. 2005) during the early generations in Cheney and Scout lakes and later generations in Loberg Lake. The Warfle Lake samples have not yet been studied. Below, we discuss major patterns of morphological and life history evolution in these recently established stickleback populations. The Loberg Lake population is the oldest and best studied population, so it is treated most extensively.

*Body shape and limnology (Figs. 2 and3A and B)*. Body shape varies substantially among Threespine Stickleback populations in relation to local environmental conditions (Walker 1997; Spoljaric and Reimchen 2007; Aguirre 2009). Oceanic stickleback have robust but streamlined bodies with large fins (Fig. 2A) adapted for swimming long distances in the open ocean (Aguirre et al. 2008; Aguirre 2009; Bell and Foster 1994a). Freshwater resident stickleback populations that they give rise to typically have smaller bodies and fins and an expanded abdominal region (Fig. 2B). The posterior tip of the pelvis and pectoral fin are shifted forward, and the caudal peduncle is elongated (Aguirre et al. 2008). Resident freshwater populations also differ substantially among themselves, with a major axis of variation ranging from benthic-feeding specialists (benthics, *sensu*McPhail 1994) at one extreme, to planktivorous specialists (limnetics, *sensu*McPhail 1994) at the other, and many populations in between (e.g., Baumgartner et al. 1988; Schluter and McPhail 1992; McPhail 1994; Walker 1997; Spoljaric and Reimchen 2007; Aguirre 2009; Willacker et al. 2010; Aguirre and Bell 2012). Benthic populations inhabit shallow lakes and mostly prey on large benthic invertebrates. They are deeper-bodied and have shorter snouts and caudal peduncles (Walker 1997; Aguirre and Bell 2012). Planktivores inhabit deep lakes with limited benthic prey and feed on zooplankton in open water. They have elongated bodies, snouts, and caudal peduncles. Freshwater stickleback are usually allopatric, but a handful of sympatric benthic–limnetic species pairs have been reported from southern British Columbia, and either ecotype may be sympatric with anadromous stickleback during the breeding season (Schluter and McPhail 1992; McPhail 1994; Gow et al. 2008).

Contemporary evolution of body shape in recently established stickleback populations was rapid after freshwater colonization, and they became indistinguishable from long-established resident freshwater populations within a few decades. Aguirre and Bell (2012) studied contemporary body shape evolution in the Loberg Lake stickleback from 1990 to 2009. Body shape in 1990, the year that the new population was discovered, resembled that of anadromous stickleback, although stable isotope data and infection with *Schistocephalus* indicate that they were born in fresh water. By 1992, the shape of the Loberg population diverged substantially from that of anadromous stickleback, occupying a location in shape space characteristic of stream and lake benthic populations. After 1992, the population evolved more slowly in the general direction of the extinct Loberg Lake population, diverging approximately 68% of the distance separating its putative ancestor and the extinct native population by 2009 (Aguirre and Bell 2012). Extending this time series through 2021 with new data shows that the population has continued to evolve steadily towards the extinct Loberg Lake population, with the means for 2020 and 2021 appearing remarkably close to the extinct population mean (Fig. 3B).

The early stickleback samples from Cheney and Scout lakes showed strikingly similar body shape divergence. Body shape means for samples from the first generation born in fresh water in each (2010 and 2012, respectively) were close to the means of their anadromous ancestor, indicating a limited effect of phenotypic plasticity, despite experimental evidence for body shape phenotypic plasticity (Wund et al. 2012; Leaver and Reimchen 2012). The populations then evolved rapidly in the direction of stream and lake benthic populations in the region, overlapping with early samples from the Loberg Lake time series. The occupation of the same portion of the body shape space early in the time series by all three recently established populations suggest that body shape divergence is predictable in YFPs.

*Lateral plate morphs (Figs. 2 and 3C)*. Lateral plates are bony defensive structures that vary greatly in number within and among stickleback populations and even within families. The phenotypes are conventionally classified as complete, partial, and low morphs (Fig. 2) based on the number and distribution of plates on the flanks (Hagen and Gilbertson 1972, 1973b; Bell 1981; Hagen and Moodie 1982; Baumgartner and Bell 1984; Bell and Foster 1994a). Oceanic populations, which develop in marine water, where plates are apparently energetically inexpensive to produce (Marchinko and Schluter 2007) and where there is no physical refuge from predatory fish (Reimchen 2000) and they presumably experience extensive fish predation but not insect predation (Marchinko 2009), are typically monomorphic for the complete morph with 30–36 (modally 33) large lateral plates and forming a keel on the caudal peduncle. Although some freshwater populations are monomorphic or polymorphic for the complete morph, the low morph predominates and is often monomorphic in fresh water along the Pacific coast of North America (Miller and Hubbs 1969; Hagen and Gilbertson 1972; Hagen and Moodie 1982; Baumgartner and Bell 1984). Lateral plates are subject to strong natural selection and are likely influenced by a number of factors (Hagen and Gilbertson 1972, 1973b; Bell 1984; Reimchen 2000; Barrett et al. 2008; Jamniczky et al. 2018). The occurrence of low morphs is common in habitats with reduced fish and increased insect predation (Reimchen 1994) and may provide a selective advantage through increased somatic growth rates in freshwater environments (Marchinko and Schluter 2007; Barrett et al. 2008) and reduced vulnerability to insect predation (Marchinko 2008). Enhanced fast-start performance has been documented in individuals with low lateral plate numbers and may also contribute to their loss (Taylor and McPhail 1986; Bergstrom 2002).

Rapid evolution of lateral plate morph frequencies has been documented in most recently established freshwater populations examined (Klepaker 1993; Bell et al. 2004, 2016; Gelmond et al. 2009; Bell and Aguirre 2013; Lescak et al. 2015; Roberts Kingman et al. 2021). In Loberg Lake, low morph stickleback appeared 1 y after the population was discovered (1991) and rapidly increased in frequency, becoming the most common morph by 1994 and increasing to a frequency of 75% by 2001 (Bell et al. 2004). Extending the time series indicates that low morphs achieved a frequency of 96% by 2017 (Fig. 3C), and complete morphs have declined to a frequency of 1%. The Cheney and Scout populations followed a similar trajectory to that of the Loberg Lake population early in the time series; low morph fish appeared within the first few years of founding and rapidly increased to frequencies of 33% in 7 y in Cheney Lake, and of 21% after 6 y in Scout Lake (Fig. 3C).

Low morph lateral plate number is the most thoroughly studied Threespine Stickleback phenotype (e.g., Miller and Hubbs 1969; Hagen and Gilbertson 1972; Reimchen 1994). It has also evolves in the Loberg Lake population (Bell 2001; Bell et al. 2004). It is associated with predation regime, with higher plate counts (means ∼7) associated with fish predation (Hagen and Gilbertson 1972; Reimchen 1994). When low morphs first appeared in Loberg Lake, they averaged 6.87 plates per side, suggesting that the ancestral low morph lateral plate number is relatively high. By 2001, mean low morph lateral plate number declined to 6.37. Unpublished data collected since 2001 show an erratic pattern of change without a clear trend. Lateral plate number in completes showed a more marked decline from 32.9 to 31.3 between 1990 and 2001.

*Gill-raker number (Fig. 3D)*. Gill raker-number and length are negatively correlated with food particle size among fish species (Magnuson and Heitz 1971) and among stickleback populations (Hagen and Gilbertson 1972; Gross and Anderson 1984). Gill-raker number is highly heritable (Hagen and Gilbertson 1972; Hermida et al. 2002; Aguirre et al. 2004) and several QTL with moderate effects for gill raker morphology have been identified on ChrIV and ChrXX, and many QTL with small effects have been found on other chromosomes, such as ChrVI, ChrXI, and ChrXIII (e.g., Miller et al. 2014; Conte et al. 2015). In stickleback, oceanic populations typically have high gill-raker counts (mean ∼22) for feeding on zooplankton (Gross and Anderson 1984; Aguirre et al. 2008), while freshwater populations can have lower (to 14) or higher (to 24) counts, depending on diet (Gross and Anderson 1984; Walker 1997).

All three of the recently established stickleback populations experienced gill-raker number reduction early in their time series. In Loberg Lake stickleback, the mean gill-raker number (22.34) of the earliest sample (1990) did not differ significantly from that (mean = 22.36) of the nearby Rabbit Slough anadromous population (Aguirre et al. 2008). It declined abruptly by 1992 (mean = 21.42) and then more gradually and irregularly thereafter (Bell et al. 2004, Rivera et al. unpublished data). Extending the time series past 2001, the gradual declining trend has continued, with several annual samples falling well below the mean of the extinct Loberg Lake population sampled in 1982 (mean = 20.94). Similarly, the mean gill-raker number was very close to that of their anadromous ancestor in the first two years of sampling in Cheney and Scout lakes. After the second year of sampling, mean gill-raker number dropped precipitously by more than one gill-raker in both populations, becoming similar to those observed in Loberg Lake later in the time series (Fig. 3D).

*Life history traits*. Body size, age at reproduction, and clutch mass and size (i.e., number of ripe eggs) in female Threespine Stickleback vary greatly among populations (Baker 1994; Baker et al. 2008) and are highly heritable (Snyder 1991). Anadromous stickleback from Rabbit Slough return to fresh water to breed after 1–4 y, but they breed most often at age 3 (Rollins et al. 2017). Even younger, smaller anadromous stickleback tend to be larger than most freshwater stickleback (Rollins et al. 2017). Variation among established freshwater populations (Baker et al. 2008) predicted evolution of lower reproductive effort (relative clutch mass), smaller clutch size, and reduced age at reproduction. Egg size varied among established populations, and evolution in young lake populations could not be predicted.

Evolution of life-history traits has been studied in the Loberg Lake population by Baker et al. (2019) and in the Cheney and Scout lake populations by Kurz et al. (2016). In Loberg Lake, reproductive effort and clutch size standardized for average female body size (standard length) declined by 28% and 41%, respectively, from ancestral values over about 21 generations in approximately 30 y. Their decline was substantial and rapid, with a strong cyclical pattern, possibly reflecting density-dependent selection. Age at reproduction among females also declined because percentages of reproducing age-1 females increased. Egg size did not change significantly.

Sampling in Loberg Lake for life history variation did not begin until several years after colonization, but the earliest samples from Cheney and Scout lakes help fill this early sampling gap. The first few years of life history variation can be complex. A high frequency of age-1 females reproduced during the first year after introduction, but female reproduction in subsequent years was mostly at age 2 (Kurz et al. 2016). Frequent breeding by age-1 females during the first generation in the lake, when age-2 females were absent, suggests that the age at which stickleback reproduce may be facultative. Age-2 females and males, which were absent from the lakes 1 y after founding, may normally suppress breeding by age-1 females. Reproductive effort and size-adjusted clutch size also increased abruptly in the first year after introduction (Kurz et al. 2016).

*Assortative mating between YFPs and the anadromous ancestor. G. aculeatus* is a species complex or superspecies (Bell 1976, 1995; Bell and Foster 1994a) that is composed mostly of diverse allopatric populations. However, it also contains pairs of sympatric, phenotypically divergent populations, including resident freshwater and anadromous populations (McPhail 1994; McKinnon and Rundle 2002). Sympatric anadromous and freshwater stickleback are phenotypically divergent (e.g., Bell and Foster 1994a), and they typically exhibit at least partial reproductive isolation in sympatry and sometimes produce fertile hybrids (Hagen 1967; Jones et al. 2006). Several factors contribute to isolation between them (McPhail 1994), and positive assortative mating is included among them (Hay and McPhail 1975). Differences in body size are particularly important for mate choice in Threespine Stickleback (McPhail 1994; Nagel and Schluter 1998; McKinnon et al. 2004; Boughman et al. 2005), and freshwater stickleback are usually much smaller than anadromous ones, providing a criterion for positive assortative mating (e.g., McPhail 1994; McKinnon et al. 2004; Karve et al. 2008).

Lake populations founded recently by anadromous stickleback quickly decline in body size. Baker et al. (2019) reported that the body length of reproductive females declined in Loberg Lake from about 71 mm in the presumptive anadromous ancestor to 45 mm in 1992, and fluctuated between approximately 45 and 48 mm for 30 y (21 estimated generations) after establishment.

Furin et al. (2012) reported mean standard lengths (SL) of about 46 mm in reproductive Loberg males and females used in 2004 and 2005 for no-choice mating trials. They observed partial reproductive isolation between Loberg Lake and anadromous Rabbit Slough stickleback. Anadromous males and Loberg females rarely mated, but anadromous females and Loberg males readily mated. This difference is consistent with male preference for larger females, which tend to have more eggs. In contrast, both sexes of Loberg Lake stickleback mated readily with stickleback from another resident freshwater population, indicating that the partial isolation was specifically between Loberg and the anadromous ancestral form. However, Rabbit Slough stickleback have two major size classes (Rollins et al. 2017), and Rabbit Slough males from the smaller size class were more likely than larger ones to mate with Loberg Lake females, suggesting that assortative mating depended strongly on body size. Sympatric reproduction of divergent anadromous and young freshwater stickleback in four lakes near Girdwood, Alaska indicates speciation of freshwater stickleback within decades after anadromous stickleback founded them (Bell and von Hippel, unpublished data).

### Contemporary genomic evolution in young freshwater populations founded recently by anadromous Threespine Stickleback

**Fig. 4:**
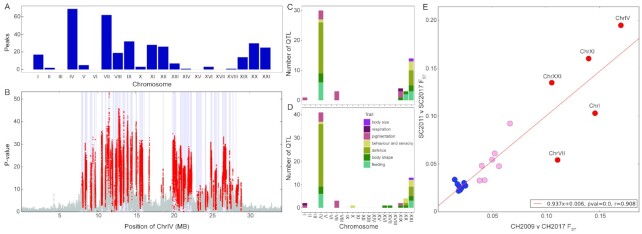
Contemporary genomic evolution in the three YFPs in Loberg, Cheney, and Scout lakes. (**A**) Numbers of rapidly increasing freshwater-adaptive peaks on the 21 chromosomes. (**B**) Cochran-Mantel-Haenszel test-statistic values for peaks of divergence across ChrIV based on combined results from the three YFPs. Note the extensive increase for large portions of ChrIV. Light blue bars mark the locations of 69 peaks of divergence between oceanic and established freshwater populations. Red indicates significant Bonferroni-corrected change in the three YFPs. (**C**) The number of significant QTL per chromosome that overlap with peak regions of evolution in the three YFPs. (**D**) The number of significant QTL per chromosome that overlap with peak regions of divergence between long-established populations in northeast Pacific lakes. (**E**) The magnitude of sequence evolution (FST) on corresponding chromosomes of the Cheney and Scout lake populations from the year of founding until 2017. Sequence divergence is highly correlated among the populations. Red, FST >0.1; pink, FST >0.03, blue, FST <0.025. Red points are for the most important chromosomes (i.e., greatest number of significantly divergent peaks) for freshwater adaptation.

Within just 5 y after introducing anadromous Threespine Stickleback to Cheney and Scout lakes (Table 1), striking shifts were observed both in the freshwater-adaptive phenotypic traits (Bell et al. 2004; Aguirre and Bell 2012; Bell et al. 2016) described above and numerous genomic regions spanning several key chromosomes (Roberts Kingman et al. 2021; Schluter et al. 2021; Fig. 4A and B). Similar phenotypic shifts were also observed in Loberg Lake samples made a few years after natural colonization (1982–88). At the genomic level, the *Eda* region matched the rapid increase of the low-plate morph (O'Brown et al. 2015; Roberts Kingman et al. 2021; Schluter et al. 2021). This shift in allele frequency at a single nucleotide polymorphism (SNP) identified as a strong candidate to be the causative SNP for low-platedness in Threespine Stickleback (O'Brown et al. 2015) was also observed in Cheney and Scout lakes, which started out at a frequency of less than 1% in both lakes at founding and was estimated to be 59.7% in Cheney and 50.6% in Scout by 2017, representing 9 and 7 y of evolution, respectively (Roberts Kingman et al. 2021).

**Fig. 5:**
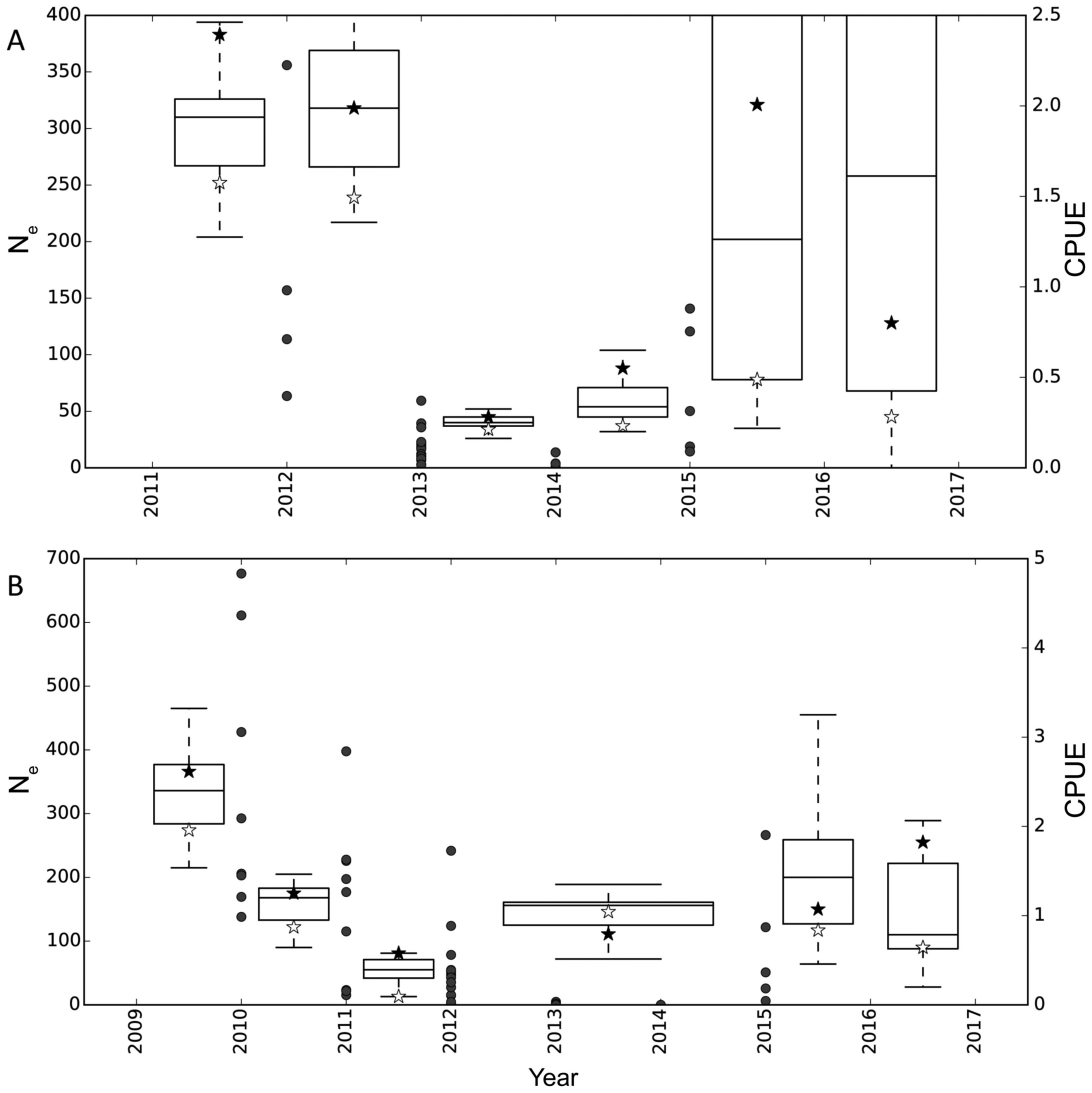
Box plots of effective population size (Ne, left y-axis) for each chromosome between successive points of the time series experiments for Scout (**A**) and Cheney lakes (**B**). Stars indicate the estimates for specific chromosomes, with black stars indicating estimates for ChrIV and white stars for ChrXV, which are the most divergent and a “neutral” chromosome, respectively. The catch per unit effort (CPUE; Bell et al. 2016) is also plotted for all samples during each year (right y-axis) with black circles.

To assess annual allele frequency changes across the Threespine Stickleback genome (∼450 Mb) in these three populations, pooled samples were sequenced (i.e., pool-seq) with ∼100 individuals per annual sample since founding of the Cheney (2009–17) and Scout (2011–17) lake populations and from 2001 to 2017 for Loberg Lake (Roberts Kingman et al. 2021). Rapid shifts in allele frequencies were observed across the genome. They were not restricted to a few candidate regions (e.g., *Eda*), but numerous significant changes in SNP frequencies were observed on most chromosomes. These significant SNPs were used to define freshwater-adaptive regions of peak divergence (haplotypes, 344 regions across 21 chromosomes, Fig. 4A) that were diverging from their anadromous ancestor in these isolates and included 17.57 Mb (∼3.73%) of the Threespine Stickleback genome (Roberts Kingman et al. 2021). Estimates of annual variation in effective population size (N_e_) showed that the Cheney and Scout populations bottlenecked 3 y after founding (Fig. 5) and bottlenecking was followed by significant increases in the frequencies of freshwater-adaptive peaks (Roberts Kingman et al. 2021).

About 97% of the rapidly evolving freshwater-adaptive regions overlapped with sequences that differ strongly between long-established freshwater and oceanic Threespine Stickleback populations in the northeastern Pacific. A smaller fraction of them are shared with freshwater populations in the Atlantic basin, which apparently were derived within the last 100 kya from Pacific basin populations and lost a substantial amount of standing genetic variation (Bell 1994, 2009; Fang et al. 2020; Roberts Kingman et al. 2021). This observation highlights the important point that standing genetic variation in the Alaskan oceanic populations is crucial for rapid adaptation in Alaskan lake populations derived recently from oceanic stickleback. Similarly, many of the same freshwater-adaptive loci diverged in freshwater populations that were founded by oceanic stickleback after the Great Alaskan Earthquake of 1964 (58 y ago) uplifted terrain on Alaskan islands where they are located (Lescak et al. 2015; Bassham et al. 2018). In contrast, however, Terekanova et al. (2014), identified substantially fewer freshwater-adaptive loci in Atlantic basin lake stickleback derived within the last several hundred years from oceanic stickleback.

Peak regions of rapid divergence were concentrated on several key chromosomes with concentrations of loci that are consistently divergent for freshwater-adaptive alleles in established freshwater populations and oceanic Threespine Stickleback (Fig. 4A, B, and E). Genetic differentiation (F_ST_) among chromosomes was estimated in the seeded lakes from the start of the experiments through 2017. Chromosomes with few to no freshwater-adaptive peaks (e.g., ChrXV) experienced little divergence, and those with substantial numbers of peaks (ChrI, ChrIV, ChrVII, and ChrXXI; Fig. 4A) showed significant differentiation from the year of founding (Fig. 4E). Several of these peak regions overlapped with known quantitative trait loci (QTL; Peichel and Marques 2017) that differ between oceanic and freshwater populations, particularly on ChrIV and ChrXXI (Fig. 4C and D). The greatest overlap was associated with defense traits (e.g., lateral plate number, plate size, spine length) on the left arm of ChrIV, which manifested significant changes in allele frequencies along most of the chromosome (Fig. 4B and C). Several QTL with large effects (i.e., % variation explained; PVE >20%) for various traits overlap at a specific region of ChrIV encompassing the *Eda* gene, which has been shown to have a strong pleiotropic effect on several freshwater adaptive traits (Albert et al. 2007; Archambeault et al. 2020). In addition to concentration of these peak regions on a few key chromosomes and overlap with known QTL, genomic architecture facilitates linkage among these regions. Many QTL for important phenotypic traits overlapped with regions of rapid evolution in recently founded populations and with regions of divergence between long-established freshwater and oceanic populations in the northeast Pacific. The estimated recombination landscape for the stickleback genome demonstrates that freshwater-adaptive peaks are often found in regions of low overall recombination rate relative to the rest of the chromosome, keeping them linked in freshwater environments. However, local recombination hotspots also occur between major divergent peaks, likely facilitating rapid disaggregation in the marine environment and reassembly after colonization of fresh water (Roberts Kingman et al. 2021), as envisioned in the transporter hypothesis (Schluter and Conte 2009). In addition, chromosome rearrangements are also associated with marine-freshwater divergence. They include three large chromosomal inversions on ChrI, ChrXI, and ChrXXI, which suppress recombination and keep regions associated with freshwater adaptation linked together (Jones et al. 2012). These regions increased rapidly in frequency in Loberg, Cheney, and Scout lakes. Similarly, chromosome fusions identified on ChrIV and ChrVII were also associated with low recombination and an increase in QTLs associated with marine-freshwater divergence (Liu et al. 2022).

Finally, compared to previous estimates of selection coefficients (*s*) from genomic data (Thurman and Barrett 2016), estimates of *s* based on allele-frequency trajectories are exceptionally high for freshwater-adaptive alleles during contemporary evolution in YFPs (Roberts Kingman et al. 2021). Individual SNPs with the highest rates of increase and greatest repeatability across populations had *s* values of 0.08 to 0.53. The estimated *s* on *Eda* in an experimental cross was 0.5 (*SD* 0.09), reflecting twice the rate of survival of F2 offspring that were homozygous for the freshwater allele compared to homozygotes for the marine allele (Schluter et al. 2021). However, simulations in Roberts Kingman et al. (2021) indicated that it is unlikely that any individual locus has such a high *s*. Rather individual SNPs have smaller *s* coefficients (i.e., gradients; *sensu*Lande and Arnold 1983) and work in concert with other advantageous SNPs at neighboring peaks (i.e., linked haplotypes) to produce larger *s* values (i.e., differentials), conflicting with Thurman and Barrett's (2016) results.

### Contemporary evolution of the genes that matter in young freshwater populations founded recently by anadromous Threespine Stickleback

Several candidate genes that are divergent between anadromous and freshwater populations have been identified. The genetic changes that underlie phenotypic evolution are generally not in protein-coding regions, but rather in sequences that influence tissue-specific regulation of gene expression. Therefore, the phenotypic changes are often for loss or reduction of phenotypic expression in the freshwater form (Colosimo et al. 2004, 2005; Cresko et al. 2004; Miller et al. 2007; O'Brown et al. 2015). A freshwater-adaptive allele of *Bmp6* is down-regulated relative to the ancestral allele, but it actually causes more robust pharyngeal teeth (Cleves et al. 2014, 2018; Erikson et al. 2015). Genes involved in phenotypic divergence between anadromous and freshwater populations are summarized in Table 2. Although several of these phenotypes have not been measured in our young populations, we assessed the overlap and proximity of the evolutionary peak regions to the genomic regions of these known genes. Genes of known function on ChrIV overlapped with identified peaks, and putative causative SNPs fell within these same peak regions. These genes control two major defense traits. *Eda* (discussed above) has a major effect on lateral plate and a myriad of other freshwater-adaptive traits. The other two genes *Msx2a* and *Stc2a* are both involved in spine length and fall within the same peak. Several studies have shown that regions important in freshwater adaptation form supergenes, where linkage imposes a strong association among these important regions (e.g., Erickson et al. 2015; Roberts Kingman et al. 2021b). The genes known to influence other diverging traits did not overlap with peaks in our experimental young populations, either because they are not rapidly evolving in them or because their regulatory sequence falls within an adjacent peak. Further studies measuring these traits in these young populations will provide more insights into the relationship between genetic function and contemporary evolution.

**Table 2:** Genes, phenotypes, and genomic regions that are divergent between oceanic and freshwater populations

			Gene position	Peak overlap	Peak distance (bp)			
Gene	Chr	Traits	Gene start	Gene end	Peak start	Peak end	Left peak	Right peak
*Eda*	IV	Plate number, lateral line	12,812,614	12,822,840	12,763,334	13,010,434		
*Msx2A*	IV	Spine length	13,918,256	13,919,508	13,858,584	14,016,284		
*stc2a*	IV	Spine length	13,942,770	13,946,215	13,858,584	14,016,284		
*Kitlg*	XIX	Pigmentation	13,883,368	13,887,611			2,556,653	627,854
*Gdf6*	XX	Plate height	15,862,030	15,867,274			1,751,160	
*Bmp6*	XXI	Pharyngeal tooth number	7,979,101	8,010,074			762,722	259,155
*Tfap2a*	XXI	Brachial bone length	8,408,774	8,421,539			139,545	100,140

The peak overlap indicates genetic regions that overlap with genes of known function, and the peak start and peak end denote the base position ranges associated with the stickleback genome GasAcu 1.4. The left peak and right peak indicate the number of base pairs away from the nearest peak is from the gene of interest, if there is no overlap between a gene and peak.

## Conclusion

Freshwater Threespine Stickleback populations have been founded innumerable times over millions of years by oceanic stickleback throughout their Holarctic range (Lindsey 1962; McPhail and Lindsey 1970; Bell 1976, 1994, 2009; Bell and Foster 1994a). The freshwater isolates diversify in relation to local conditions and undergo adaptive radiation (e.g., Hagen and Gilbertson 1972; Bell 1976, 1984; Campbell 1985; Reimchen et al. 1985; Spoljaric and Reimchen 2007).They evolve a set of convergent phenotypic traits that are absent from their oceanic ancestors (Bell 1976; 1984). Although these populations are isolated within countless, separate drainages, the alleles responsible for their convergent evolution are shared by common ancestry (DeFaveri et al. 2011; Jones et al. 2012; Roberts Kingman et al. 2021). Hundreds of these freshwater-adaptive alleles are carried by oceanic populations as standing genetic variation that is quickly assembled by strong natural selection after colonization of fresh water into a genome that encodes typical freshwater phenotypes (Schluter and Conte 2009; Jones et al. 2012; Lescak et al. 2015; Magalhaes et al. 2020; Roberts Kingman et al. 2021). Remarkably, our results show that much of the characteristic phenotypic and genomic divergence between ancestral oceanic and their freshwater descendants can evolve within the first few decades after freshwater populations are derived from oceanic ancestors.

New freshwater populations continue to be founded naturally by oceanic stickleback (Bell and Aguirre 2013), and they can be established experimentally (Terekhanova et al. 2014; Bell et al. 2016). These young freshwater isolates evolve very rapidly, as we show, and this phenomenon enables acquisition of unique insights into evolutionary dynamics. Combined with our exceptionally deep and broad knowledge of Threespine Stickleback biology (Wootton 1976, 1984; Ziuganov 1991; Bell and Foster 1994a; Paepke 1996; Östland-Nilsson et al. 2007; von Hippel 2010) and excellent molecular genetic and genomic tools (Kingsley and Peichel 2007; Peichel and Marquez 2017; Reid et al. 2021), these YFPs provide exceptional opportunities to study the evolutionary dynamics of phenotypes, genes, and genomic architecture (Roberts Kingman et al. 2021). They also provide the material to investigate general issues in evolutionary molecular genetics and genomics.

Ten YFPs on the Kenai Peninsula or within the Cook Inlet basin, Alaska, vary for the time since they were founded by anadromous stickleback (Table 1). They are readily accessible and offer excellent opportunities to study the genetic and genomic foundations for phenotypic divergence and speciation. Freshwater habitats on Alaskan islands originated after 1964 and offer similar opportunities but are less accessible (Gelmond et al. 2009; Lescak et al. 2015; Bassham et al. 2018). We plan to continue to make annual samples from Loberg, Cheney, Scout, and “Warfle” lakes and possibly other young lake populations. We will extend phenotypic time series through at least 2022 to infer whether morphological divergence is consistent among populations and will compare them and the other young populations to infer whether divergence of our latest samples from each population from their presumptive anadromous ancestors have followed a common time course. Comparison of morphological outcomes combined with genomic divergence in these 10 young populations can provide insights into the influence of genetic constraint (Connallon and Hall 2018) on the evolutionary response of phenotypes, genes, and genomes to selection. In addition, advanced genomic approaches are now more readily available and implementable, allowing for the investigation of which traits these diverging regions influence as well as the epigenomic landscape, which may be important in influencing adaptation in the early years after founding, before allele frequencies change.

Three YFPs in impoundments at miles 85, 87, and 88 of Seward Highway, near Girdwood, Alaska must have formed nearly simultaneously and open to Cook Inlet within 5 km of each other (Table 1). They were probably founded roughly simultaneously since 1964 by a single anadromous population, with which they still breed in sympatry but from which they are sufficiently isolated to diverge for many phenotypic traits (Bell and von Hippel, unpublished data). Their founders were finite populations drawn from a single ancestral population and must have experienced genetic drift independently and may experience somewhat different ecological conditions. They are nearly replicate populations that we will compare closely. In addition, unlike other YFPs in Cook Inlet, they breed in sympatry with anadromous populations, with which they may still hybridize, and the effects and levels of gene flow on divergence, selection against freshwater adaptive alleles in anadromous populations, and isolating mechanisms can be studied.

Since Hendry and Kinnison (1999) drew attention to contemporary evolution, studies of this theoretically important phenomenon have proliferated (Hendry et al. 2008, Sanderson et al. 2022). However, substantial contemporary evolution is difficult to recognize and probably far more common than appreciated. It is usually discovered serendipitously during unrelated research. For example, Bell (2001) discovered contemporary evolution in the Loberg Lake stickleback population while sampling for research on pelvic girdle reduction (Bell et al. 1993; Bell and Ortí 1994). Even when contemporary evolution is detected, only two or a few generations are usually sampled (Hendry et al. 2008). Samples must be made frequently enough and for long enough to estimate true rates of evolution and to resolve patterns of change through time (Gingerich 2019). Thus, investigators should be vigilant for opportunities to study contemporary evolution and, without assurance of success, make periodic samples at fine enough intervals to resolve evolutionary patterns.

## Notes

From the symposium The deep and shallow history of aquatic life's passages between marine and freshwater habitats presented at the annual meeting of the Society for Integrative and Comparative Biology, January 7, 2022 at Phoenix, Arizona.

## References

[bib1] Aguirre WE. 2009. Microgeographical diversification of Threespine Stickleback: body shape-habitat correlations in a small, ecologically diverse Alaskan drainage. Biol J Linn Soc. 98: 139–51.

[bib2] Aguirre WE , Bell MA. 2012. Twenty years of body shape evolution in a Threespine Stickleback population adapting to a lake environment. Biol J Linn Soc. 105: 817–31.

[bib3] Aguirre WE , Doherty PK, Bell MA. 2004. Genetics of lateral plate and gill raker phenotypes in a rapidly evolving population of Threespine Stickleback. Behaviour. 141: 1465–83.

[bib4] Aguirre WE , Ellis KE, Kusenda M, Bell MA. 2008. Phenotypic variation and sexual dimorphism in anadromous threespine stickleback: implications for postglacial adaptive radiation. Biol J Linn Soc. 95: 465–78.

[bib5] Albert AYK , Sterling S, Vines TH, Knecht AK, Miller CT, Summers BR, Balabhadra S, Kingsley DM, Schluter D. 2007. The genetics of adaptive shape shift in stickleback: pleiotropy and effect size. Evolution. 62: 76–85. 18005154 10.1111/j.1558-5646.2007.00259.x

[bib6] Archambeault SL , Bärtschi LR, Merminod AD, Peichel CL. 2020. Adaptation via pleiotropy and linkage: association mapping reveals a complex genetic architecture within the stickleback *Eda* locus. Evol Lett. 4: 282–301. 32774879 10.1002/evl3.175 PMC7403726

[bib7] Arif S , Aguirre WE, Bell MA. 2009. Evolutionary diversification of operculum shape in Cook Inlet Threespine Stickleback. Biol J Linn Soc. 97: 832–44.

[bib8] Baker JA. 1994. Life history variation in female Threespine Stickleback. In: Bell MA, Foster SA, editors. The evolutionary biology of the Threespine Stickleback. Oxford: Oxford University Press. p. 144–87.

[bib9] Baker JA , Heins DC, Foster SA, King RW. 2008. An overview of life-history variation in female Threespine Stickleback. Behaviour. 145: 579–602.

[bib10] Baker JA , Heins DC, Baum JE. 2019. Trajectory and rate of change in female life-history traits following colonization of a freshwater, lacustrine environment by oceanic Threespine Stickleback. Evol Ecol Res. 20: 247–63.

[bib11] Barrett RD , Schluter D. 2008. Adaptation from standing genetic variation. Trends Ecol Evol. 23: 38–44. 18006185 10.1016/j.tree.2007.09.008

[bib12] Barrett RDH , Rogers SM, Schluter D. 2008. Natural selection on a major armor gene in Threespine Stickleback. Science. 322: 255–7. 18755942 10.1126/science.1159978

[bib13] Bassham S , Catchen J, Lescak E, von Hippel FA, Cresko WA. 2018. Repeated selection of alternatively adapted haplotypes creates sweeping genomic remodeling in stickleback. Genetics. 209: 921–39. 29794240 10.1534/genetics.117.300610 PMC6028257

[bib14] Bateson W. 1900. Collective enquiry as to progressive melanism in moths—memorandum from the Evolution Committee of the Royal Society. Entomol Rec. 12: 140.

[bib15] Baumgartner JV , Bell MA. 1984. Lateral plate morph variation in California populations of the Threespine Stickleback, *Gasterosteus aculeatus*. Evolution. 38: 665–74. 28555988 10.1111/j.1558-5646.1984.tb00333.x

[bib16] Baumgartner JV , Bell MA, Weinberg PH. 1988. Body form differences between the Enos Lake species pair of Threespine Stickleback (*Gasterosteus aculeatus* complex). Can J Zool. 66: 467–74.

[bib17] Bell MA. 1976. Evolution of phenotypic diversity in the *Gasterosteus aculeatus* superspecies on the Pacific coast of North America. Syst Zool. 25: 211–27.

[bib18] Bell MA. 1981. Lateral plate polymorphism and ontogeny of the complete plate morph of Threespine Sticklebacks (*Gasterosteus aculeatus*). Evolution. 35: 67–74. 28563462 10.1111/j.1558-5646.1981.tb04859.x

[bib19] Bell MA. 1984. Evolutionary phenetics and genetics. The Threespine Stickleback, *Gasterosteus aculeatus* and related species. In: Turner BJ, editor. Evolutionary genetics of fishes. New York (NY): Plenum Press. p. 431–528.

[bib20] Bell MA. 1994. Paleobiology and evolution of Threespine Stickleback. In: Bell MA, Foster SA, editors. The evolutionary biology of the Threespine Stickleback. Oxford: Oxford University Press. p. 439–71.

[bib21] Bell MA. 1995. Intraspecific systematics of *Gasterosteus aculeatus* populations: implications for behavioral ecology. Behaviour. 132: 1131–52.

[bib22] Bell MA. 2001. Lateral plate evolution in the Threespine Stickleback: getting nowhere fast. Genetica. 112/113: 445–61. 11838781

[bib23] Bell MA. 2009. Implications of fossil Threespine Stickleback for Darwinian gradualism. J Fish Biol. 75: 1977–99. 20738668 10.1111/j.1095-8649.2009.02416.x

[bib24] Bell MA , Aguirre WE. 2013. Contemporary evolution, allelic recycling, and adaptive radiation of the Threespine Stickleback. Evol Ecol Res. 15: 377–411.

[bib25] Bell MA , Foster SA. 1994a. Introduction to the evolutionary biology of the Threespine Stickleback. In: Bell MA, Foster SA, editors. The evolutionary biology of the Threespine Stickleback. Oxford: Oxford University Press. p. 1–27.

[bib26] Bell MA, Foster SA, (eds).1994b.The evolutionary biology of the Threespine Stickleback. Oxford: Oxford University Press.

[bib27] Bell MA , Ortí G. 1994. Pelvic reduction in Threespine Stickleback from Cook Inlet lakes: geographic distribution and intrapopulation variation. Copeia. 1994: 314–25.

[bib28] Bell MA , Aguirre WE, Buck NJ. 2004. Twelve years of contemporary armor evolution in a Threespine Stickleback population. Evolution. 58: 814–24. 15154557 10.1111/j.0014-3820.2004.tb00414.x

[bib29] Bell MA , Gangavalli A, Bewick A, Aguirre WE. 2010. Frequency of *Ectodysplasin* alleles and limited introgression between sympatric Threespine Stickleback populations. Environ Biol Fishes. 89: 189–98.

[bib30] Bell MA , Heins DC, Wund MA, von Hippel FA, Massengill R, Dunker K, Bristow GA, Aguirre WE. 2016. Reintroduction of Threespine Stickleback into Cheney and Scout Lakes, Alaska. Evol Ecol Res. 17: 157–78.

[bib31] Bell MA , Ortí G, Walker JA, Koenings JP. 1993. Evolution of pelvic reduction in Threespine Stickleback fish: a test of competing hypotheses. Evolution. 47: 906–14. 28567888 10.1111/j.1558-5646.1993.tb01243.x

[bib32] Bell MA , Stewart JD, Park PJ. 2009. The world's oldest fossil Threespine Stickleback. Copeia. 2009: 256–65.

[bib33] Bergstrom CA. 2002. Fast-start swimming performance and reduction in lateral plate number in Threespine Stickleback. Can J Zool. 80: 207–13.

[bib34] Boughman JW , Rundle HD, Schluter D. 2005. Parallel evolution of sexual isolation in sticklebacks. Evolution. 59: 361–73. 15807421

[bib35] Brakefield PM , Liebert TG. 2000. Evolutionary dynamics of declining melanism in the peppered moth in the Netherlands. Proc R Soc Lond B Biol Sci. 267: 1953–7. 10.1098/rspb.2000.1235 PMC1690762 11075707

[bib36] Campbell RN. 1985. Morphological variation in the three-spined stickleback (*Gasterosteus aculeatus*) in Scotland. Behaviour. 93: 161–8.

[bib37] Cleves PA , Ellis NA, Jimenez MT, Nunez SM, Schluter D, Kingsley DM, Miller CT. 2014. Evolved tooth gain in sticklebacks is associated with a cis-regulatory allele of *Bmp6*. Proc Natl Acad Sci. 111: 13912–7. 25205810 10.1073/pnas.1407567111 PMC4183278

[bib38] Cleves PA , Hart JC, Agoglia RM, Jimenez MT, Erickson PA, Gai L, Miller CT. 2018. An intronic enhancer of *Bmp6* underlies evolved tooth gain in sticklebacks. PLoS Genet. 14: e1007449. 29902209 10.1371/journal.pgen.1007449 PMC6019817

[bib39] Colosimo PF , Peichel CL, Nereng K, Blackman BK, Shapiro MD, Schluter D, Kingsley DM. 2004. The genetic architecture of parallel armor plate reduction in Threespine Sticklebacks. PLoS Biol. 2: 635–41. 10.1371/journal.pbio.0020109 PMC385219 15069472

[bib40] Colosimo PF , Hosemann KE, Balabhadra S, Villarreal G, Dickson M, Grimwood J, Schmutz J, Myers RM, Schluter D, Kingsley DM. 2005. Widespread parallel evolution in sticklebacks by repeated fixation of *Ectodysplasin* alleles. Science. 307: 1928–33. 15790847 10.1126/science.1107239

[bib41] Connallon T , Hall MD. 2018. Genetic constraints on adaptation: a theoretical primer for the genomics era. Ann NY Acad Sci. 1422: 65–87. 29363779 10.1111/nyas.13536

[bib42] Conte GL , Arnegard ME, Best J, Chan YF, Jones FC, Kingsley DM, Schluter D, Peichel CL. 2015. Extent of QTL reuse during repeated phenotypic divergence of sympatric Threespine Stickleback. Genetics. 201: 1189–200. 26384359 10.1534/genetics.115.182550 PMC4649644

[bib43] Cook LM , Sacchari IJ. 2013. The peppered moth and industrial melanism: evolution of a natural selection case study. Heredity. 110: 207–12. 23211788 10.1038/hdy.2012.92 PMC3668657

[bib44] Cresko WA , Amores A, Wilson C, Murphy J, Currey M, Phillips P, Bell MA, Kimmel C, Postlethwait J. 2004. The genetic basis of recurrent evolution: armor loss in Alaskan populations of Threespine Stickleback, *Gasterosteus aculeatus*. Proc Natl Acad Sci. 101: 6050–5. 15069186 10.1073/pnas.0308479101 PMC395921

[bib45] Darwin C R. 1859. The origin of species by means of natural selection or preservation of favoured races in the struggle for life. (Avenal 1979 reprint of the first edition.). New York (NY): Avenal Books.

[bib46] Darwin Correspondence Project , “Letter no. 1705”. https://www.darwinproject.ac.uk/letter/?docId = letters/DCP-LETT-1705.xml. Last accessed on January 23, 2022.

[bib47] DeFaveri J , Shikano T, Shimada Y, Goto A, Merilä J. 2011. Global analysis of genes involved in freshwater adaptation in Threespine Sticklebacks (*Gasterosteus aculeatus*). Evolution. 65: 1800–7. 21644964 10.1111/j.1558-5646.2011.01247.x

[bib48] Erickson PA , Cleves PA, Ellis NA, Schwalbach KT, Hart JC, Miller CT. 2015. A 190 base pair, TGF-β responsive tooth and fin enhancer is required for stickleback *Bmp6* expression. Dev Biol. 401: 310–23. 25732776 10.1016/j.ydbio.2015.02.006 PMC4424077

[bib49] Fang B , Kemppainen P, Momigliano P, Feng X, Merilä J. 2020. On the causes of geographically heterogeneous parallel evolution in sticklebacks. Nat Ecol Evol. 4: 1105–15. 32572218 10.1038/s41559-020-1222-6

[bib50] Fisher RA. 1930. The genetical theory of natural selection. Oxford: Clarendon Press.

[bib51] Francis RC , Havens AC, Bell MA. 1985. Unusual lateral plate variation of Threespine Sticklebacks (*Gasterosteus aculeatus*) from Knik Lake, Alaska. Copeia, 1985: 619–24.

[bib52] Furin CG , von Hippel FA, Bell MA. 2012. Partial reproductive isolation of a recently derived resident-freshwater population of Threespine Stickleback. (*Gasterosteus aculeatus*) from its putative anadromous ancestor. Evolution. 66: 3277–86. 23025615 10.1111/j.1558-5646.2012.01672.x PMC3464953

[bib53] Garcia-Elfring A , Paccard A, Thurman TJ, Wasserman BA, Palkovacs EP, Hendry AP, Barrett RD. 2021. Using seasonal genomic changes to understand historical adaptation to new environments: parallel selection on stickleback in highly-variable estuaries. Mol Ecol. 30: 2054–64. 33713378 10.1111/mec.15879

[bib54] Gelmond O , von Hippel FA, Christy MS. 2009. Rapid ecological speciation in three-spined stickleback from Middleton Island, Alaska: the roles of selection and geographical isolation. J Fish Biol. 75: 2037–51. 20738670 10.1111/j.1095-8649.2009.02417.x

[bib55] Gibson G. 2005. The synthesis and evolution of a supermodel. Science. 307: 1890–1. 15790836 10.1126/science.1109835

[bib56] Gingerich PD. 2019. Rates of evolution: a quantitative synthesis. Cambridge: Cambridge University Press.

[bib57] Gould SJ , Vrba ES. 1982. Exaptation-A missing term in the science of form. Paleobiology. 8: 4–15.

[bib58] Gow JL , Rogers SM, Jackson M, Schluter D. 2008. Ecological predictions lead to the discovery of a benthic–limnetic sympatric species pair of Threespine Stickleback in Little Quarry Lake, British Columbia. Can J Zool. 86: 564–71.

[bib59] Grant PR , Grant RB. 2002. Unpredictable evolution in a 30-year study of Darwin's finches. Science. 296: 707–11. 11976447 10.1126/science.1070315

[bib60] Greenwood AK , Mills MG, Wark AR, Archambeault SL, Peichel CL. 2016. Evolution of schooling behavior in Threespine Sticklebacks is shaped by the *Eda* gene. Genetics. 203: 677–81. 27052567 10.1534/genetics.116.188342 PMC4896186

[bib61] Gross HP , Anderson JM. 1984. Geographic variation in the gillrakers and diet of European Threespine Sticklebacks, *Gasterosteus aculeatus*. Copeia, 1984: 87–97.

[bib62] Hagen DW. 1967. Isolating mechanisms in Threespine Sticklebacks (*Gasterosteus*). J Fish Res Board Can. 24: 1637–92.

[bib63] Hagen DW , Gilbertson LG. 1972. Geographic variation and environmental selection in *Gasterosteus aculeatus* L. in the Pacific Northwest, America. Evolution. 26: 32–51. 28555771 10.1111/j.1558-5646.1972.tb00172.x

[bib64] Hagen DW , Gilbertson LG. 1973. Selective predation and the intensity of selection acting upon the lateral plates of Threespine Sticklebacks. Heredity. 30: 273–87.

[bib65] Hagen DW , Gilbertson LG. 1973. The genetics of plate morphs in freshwater Threespine Sticklebacks. Heredity. 31: 75–84.

[bib66] Hagen DW , Moodie GEE. 1982. Polymorphism for plate morphs in *Gasterosteus aculeatus* on the east coast of Canada and an hypothesis for their global distribution. Can J Zool. 60: 1032–42.

[bib67] Hay DE , McPhail JD. 1975. Mate selection in Threespine Sticklebacks (*Gasterosteus*). Can J Zool. 53: 441–50.

[bib68] Hendry AP , Kinnison MT. 1999. The pace of modern life: measuring rates of contemporary microevolution. Evolution. 53: 1637–53. 28565449 10.1111/j.1558-5646.1999.tb04550.x

[bib69] Hendry AP , Farrugia TJ, Kinnison MT. 2008. Human influences on rates of phenotypic change in wild animal populations. Mol Ecol. 17: 20–9. 18173498 10.1111/j.1365-294X.2007.03428.x

[bib70] Hermida M , Fernández C, Amaro R, San Miguel E. 2002. Heritability and “evolvability” of meristic characters in a natural population of *Gasterosteus aculeatus*. Can J Zool. 80: 532–41.

[bib71] Hermisson J , Pennings PS. 2005; Molecular population genetics of adaptation from standing genetic variation. Genetics. 169: 2335–52. 15716498 10.1534/genetics.104.036947 PMC1449620

[bib72] Jamniczky HA , Le A, Barry TN, Rogers SM. 2018. Freshwater influence is associated with differences in bone mineral density and armour configuration in Threespine Stickleback (*Gasterosteus aculeatus*). Facets. 3: 665–81.

[bib73] Jones F , Brown C, Pemberton J, Braithwaite V. 2006. Reproductive isolation in a Threespine Stickleback hybrid zone. J Evol Biol. 19: 1531–44. 16910983 10.1111/j.1420-9101.2006.01122.x

[bib74] Jones FC , Grabherr MG, Chan YF, Russell P, Mauceli E, Johnson J, Swofford R, Pirun M, Zody MC, White S et al. 2012. The genomic basis of adaptive evolution in Threespine Sticklebacks. Nature. 484 : 55–61. 22481358 10.1038/nature10944 PMC3322419

[bib75] Karve AD , von Hippel FA, Bell MA. 2008. Isolation between sympatric anadromous and resident Threespine Stickleback species in Mud Lake, Alaska. Environ Biol Fishes. 81: 287–96.

[bib76] Kawahara R , Miya M, Mabuchi K, Near TJ, Nishida M. 2009. Stickleback phylogenies resolved: evidence from mitochondrial genomes and 11 nuclear genes. Mol Phylogenet Evol. 50: 401–4. 19027080 10.1016/j.ympev.2008.10.014

[bib77] Kettlewell B. 1973. The evolution of melanism. The study of recurring necessity; with special reference to industrial melanism in the Lepidoptera. Oxford: Clarendon Press.

[bib78] Kingsley DM , Peichel CL. 2007. The molecular genetics of evolutionary change in sticklebacks. In: Östland-Nilsson S, Mayer I, Huntingford FA, editors. Biology of the three-spined stickleback. Boca Raton (FL): CRC Press. p. 42–81.

[bib79] Klepaker T. 1993. Morphological changes in a marine population of threespined stickleback, *Gasterosteus aculeatus*, recently isolated in fresh water. Can J Zool. 71: 1251–8.

[bib80] Kurz ML , Heins DC, Bell MA, von Hippel FA. 2016. Shifts in life-history traits of two introduced populations of Threespine Stickleback. Evol Ecol Res. 17: 225–42.

[bib81] Lamichhaney S , Han F, Webster MT, Andersson L, Grant BR, Grant PR. 2018. Rapid hybrid speciation in Darwin's finches. Science. 359: 224–8. 29170277 10.1126/science.aao4593

[bib82] Lande R , Arnold SJ. 1983. The measurement of selection on correlated characters. Evolution. 37: 1210–26. 28556011 10.1111/j.1558-5646.1983.tb00236.x

[bib83] Leaver SD , Reimchen TE. 2012. Abrupt changes in defence and trophic morphology of the giant Threespine Stickleback (*Gasterosteus* sp.) following colonization of a vacant habitat. Biol J Linn Soc. 107: 494–509.

[bib84] Lescak EA , Bassham SL, Catchen J, Gelmond O, Sherbick ML, von Hippel FA, Cresko WA. 2015. Evolution of stickleback in 50 years on earthquake-uplifted islands. Proc Natl Acad Sci. 112: E7204–12. 26668399 10.1073/pnas.1512020112 PMC4702987

[bib85] Lindsey CC. 1962. Experimental study of meristic variation in a population of Threespine Stickleback, *Gasterosteus aculeatus*. Can J Zool. 40: 271–312.

[bib135_1655738395893] Liu Z , Roesti M, Marques D, Hiltbrunner M, Saladin V, Peichel CL. 2022. Chromosomal fusions facilitate adaptation to divergent environments in threespine stickleback. Mol Biol Evol. 39:msab358. 34908155 10.1093/molbev/msab358 PMC8826639

[bib92] McKinnon JS , Rundle HD. 2002. Speciation in nature: the Threespine Stickleback model systems. Trends Ecol Evol. 17: 480–8.

[bib93] McKinnon JS , Mori S, Blackman BK, David L, Kingsley DM, Jamieson L, Chou J, Schluter D. 2004. Evidence for ecology's role in speciation. Nature. 429: 294–8. 15152252 10.1038/nature02556

[bib94] McNeilly T. 1968. Evolution in closely adjacent plant populations. III. *Agrostis tenuis* on a small copper mine. Heredity. 23: 99–108.

[bib95] McPhail JD. 1994. Speciation and the evolution of reproductive isolation in the sticklebacks (*Gasterosteus*) of south–western British Columbia. In: Bell MA, Foster SA, editors. The evolutionary biology of the Threespine Stickleback. Oxford: Oxford University Press. p. 399–437.

[bib96] McPhail JD , Lindsey CC. 1970. Freshwater fishes of northwestern Canada and Alaska. Bull Fish Res Bd Can. 173: 1–381.

[bib86] Magalhaes IS , Whiting JR, D'Agostino D, Hohenlohe PA, Mahmud M, Bell MA, Skúlason S, McColl ADC. 2021. Intercontinental genomic parallelism in multiple three-spined stickleback adaptive radiations. Nat Ecol Evol. 5: 251–61. 33257817 10.1038/s41559-020-01341-8 PMC7858233

[bib87] Magnuson JJ , Heitz JG. 1971. Gill raker apparatus and food selectivity among mackerels, tunas, and dolphins. Fish Bull. 69: 361–70.

[bib88] Majerus MEN. 2009. Industrial melanism in the peppered moth, *Biston betularia*: an excellent teaching example of Darwinian evolution in action. Evolution: Education and Outreach. 2: 63–74.

[bib89] Marchinko KB. 2009. Predition's role in repeated phenotypic and genetic divergence of armor in Threespine Stickleback. Evolution. 63: 127–38. 18803682 10.1111/j.1558-5646.2008.00529.x

[bib90] Marchinko KB , Schluter D. 2007. Parallel evolution by correlated response: lateral plate reduction in Threespine Stickleback. Evolution. 61: 1084–90. 17492963 10.1111/j.1558-5646.2007.00103.x

[bib91] Matuszewski S , Hermisson J, Kopp M. 2015. Catch me if you can: adaptation from standing genetic variation to a moving phenotypic optimum. Genetics. 200: 1255–74. 26038348 10.1534/genetics.115.178574 PMC4574244

[bib97] Miller CT , Beleza S, Pollen AA, Schluter D, Kittles RA, Shriver MD, Kingsley DM. 2007. Cis-regulatory changes in *Kit ligand*expression and parallel evolution of pigmentation in sticklebacks and humans. Cell. 131: 1179–89. 18083106 10.1016/j.cell.2007.10.055 PMC2900316

[bib98] Miller CT , Glazer AM, Summers BR, Blackman BK, Norman AR, Shapiro MD, Cole BL, Peichel CL, Schluter D, Kingsley DM. 2014. Modular skeletal evolution in sticklebacks is controlled by additive and clustered quantitative trait loci. Genetics. 197: 405–20. 24652999 10.1534/genetics.114.162420 PMC4012497

[bib99] Miller RR , Hubbs CL. 1969. Systematics of *Gasterosteus aculeatus*, with particular reference to intergradation and introgression along the Pacific coast of North America: a commentary on a recent contribution. Copeia. 1969: 52–69.

[bib100] Mills MG , Greenwood AK, Peichel CL. 2014. Pleiotropic effects of a single gene on skeletal development and sensory system patterning in sticklebacks. EvoDevo. 5: 5. 24499504 10.1186/2041-9139-5-5 PMC3976036

[bib101] Nagel L , Schluter D. 1998. Body size, natural selection, and speciation in sticklebacks. Evolution. 52: 209–18. 28568156 10.1111/j.1558-5646.1998.tb05154.x

[bib102] Nelson TC , Cresko WA. 2018. Ancient genomic variation underlies repeated ecological adaptation in young stickleback populations. Evol Letters. 2: 9–21. 10.1002/evl3.37 PMC6121857 30283661

[bib103] O'Brown NM , Summers BR, Jones FC, Brady DS, Kingsley DM. 2015. A recurrent regulatory change underlying altered expression and *Wnt* response of the stickleback armor plates gene *EDA*. eLife. 4: e05290. 25629660 10.7554/eLife.05290 PMC4384742

[bib104] Orr HA. 1998. The population genetics of adaptation: the distribution of factors fixed during adaptive evolution. Evolution. 52: 935–49. 28565213 10.1111/j.1558-5646.1998.tb01823.x

[bib105] Östland-Nilsson S, Mayer I, Huntingford FA, editors. 2007; Biology of the Three-spined Stickleback. Boca Raton (FL): CRC Press.

[bib106] Paepke H-J. 1996. Die stichlinge: Gasterosteidae. Vol. 10, Magdeburg: Die neue Brehm-Bücherei*Bd*.

[bib107] Peichel CL , Marques D A. 2017. The genetic and molecular architecture of phenotypic diversity in sticklebacks. Philos Trans R Soc B: Biol Sci. 372: 20150486. 10.1098/rstb.2015.0486 PMC5182418 27994127

[bib108] Reid K , Veeramah KR, Bell MA. 2021. Threespine Stickleback: a model system for evolutionary genomics. Annu Rev Genomics Hum Genet. 22: 357–83. 33909459 10.1146/annurev-genom-111720-081402 PMC8415275

[bib109] Reimchen TE. 1994. Predators and morphological evolution in Threespine Stickleback. In: Bell MA, Foster SA, editors. The evolutionary biology of the Threespine Stickleback. Oxford: Oxford University Press. p. 240–76.

[bib110] Reimchen TE. 2000. Predator handling failures of lateral plate morphs in *Gasterosteus aculeatus*: functional implications for the ancestral plate condition. Behaviour. 137: 1081–96.

[bib111] Reimchen TE , Stinson EM, Nelson JS. 1985. Multivariate differentiation of parapatric and allopatric populations of Threespine Stickleback in the Sangan river watershed, Queen Charlotte Islands. Can J Zool. 63: 2944–51.

[bib112] Roberts Kingman GA , Vyas DN, Jones FC, Brady SD, Chen HI, Reid K, Milhaven M, Bertino TS, Aguirre WE, Heins DC et al. 2021. Predicting future from past: the genomic basis of recurrent and rapid stickleback evolution. Sci Adv. 7: eabg5285. 34144992 10.1126/sciadv.abg5285 PMC8213234

[bib113] Rollins JL , Chiang P, Waite JN, von Hippel FA, Bell MA. 2017. Jacks and Jills: alternative life history phenotypes and skewed sex ratio in anadromous Threespine Stickleback (*Gasterosteus aculeatus*). Evol Ecol Res. 18: 363–82.

[bib136_1655739517327] Sanderson S , Beausoleil M, O’Dea RE, Wood ZT, Correa C, Frankel V, Gorné LD, Haines GE, Kinnison MT, Oke KB et al. 2022. The pace of modern life, revisited. Mol Ecol. 31: 1028–43. 34902193 10.1111/mec.16299

[bib115] Schluter D , Conte GL. 2009. Genetics and ecological speciation. Proc Natl Acad Sci. 106 : 9955–62. 19528639 10.1073/pnas.0901264106 PMC2702799

[bib116] Schluter D , McPhail JD. 1992. Ecological character displacement and speciation in sticklebacks. Am Nat. 140: 85–108. 19426066 10.1086/285404

[bib117] Schluter D , Marchinko KB, Arnegard ME, Zhang H, Brady SD, Jones FC, Bell MA, Kingsley DM. 2021. Fitness maps to a large-effect locus in introduced stickleback populations. Proc Natl Acad Sci. 118: e1914889118. 33414274 10.1073/pnas.1914889118 PMC7826376

[bib118] Snyder RJ. 1991. Quantitative genetic analysis of life histories in two freshwater populations of the Threespine Stickleback. Copeia. 1991: 526–9.

[bib119] Spoljaric MA , Reimchen TE. 2007. 10 000 years later: evolution of body shape in Haida Gwaii three-spined stickleback. J Fish Biol. 70: 1484–503.

[bib120] Stuart YE , Campbell TS, Hohenlohe PA, Reynolds RG, Revell LJ, Losos JB. 2014. Rapid evolution of a native species following invasion by a congener. Science. 346: 463–6. 25342801 10.1126/science.1257008

[bib121] Taylor EB , McPhail JD. 1986. Prolonged and burst swimming in anadromous and freshwater Threespine Stickleback, *Gasterosteus aculeatus*. Can J Zool. 64: 416–20.

[bib122] Taylor EB , McPhail JD. 1999. Evolutionary history of an adaptive radiation in species pairs of Threespine Sticklebacks (*Gasterosteus*): insights from mitochondrial DNA. Biol J Linn Soc. 66: 271–91.

[bib123] Taylor B , McPhail JD. 2000. Historical contingency and determinism interact to prime speciation in sticklebacks. Proc R Soc Lond B Biol Sci. 267: 2375–84. 10.1098/rspb.2000.1294 PMC1690834 11133026

[bib124] Terekhanova NV , Logacheva MD, Penin AA, Neretina TV, Barmintseva AE, Bazykin GA, Kondrashiv AS, Mugue NS. 2014. Fast evolution from precast bricks: genomics of young freshwater populations of Threespine Stickleback *Gasterosteus aculeatus*. PLoS Genet. 10: e1004696. 25299485 10.1371/journal.pgen.1004696 PMC4191950

[bib125] Thurman TJ , Barrett RDH. 2016. The genetic consequences of selection in natural populations. Mol Ecol. 25: 1429–48. 26836758 10.1111/mec.13559

[bib134_1655726999096] Varadharajan S , Rastas P, Löytynoja A, Matschiner M, Calboli FCF, Guo B, Nederbragt AJ, Jakobsen KS, Merilä J. 2019. A high-quality assembly of the nine-spined stickleback (Pungitius pungitius) genome. GBE. 11:3291–308. 31687752 10.1093/gbe/evz240 PMC7145574

[bib126] von Hippel FA. 2010. Tinbergen's legacy in behavior. Leiden: Brill.

[bib127] von Hippel FA , Weigner H. 2004. Sympatric-anadromous resident pairs of Threespine Stickleback species in young lakes and streams at Bering Glacier, Alaska. Behaviour. 141: 1441–64.

[bib128] Walker JA. 1997. Ecological morphology of lacustrine Threespine Stickleback *Gasterosteus aculeatus* L. (Gasterosteidae) body shape. Biol J Linn Soc. 61: 3–50.

[bib129] Willacker JJ , Hippel FA, PR Wilton, Walton KM. 2010. Classification of Threespine Stickleback along the benthic–limnetic axis. Biol J Linn Soc. 101: 595–608. 10.1111/j.1095-8312.2010.01531.x PMC3017379 21221422

[bib130] Withler RE , McPhail JD. 1985. Genetic variability in freshwater and anadromous sticklebacks (*Gasterosteus aculeatus*) of southern British Columbia. Can J Zool. 63: 528–33.

[bib131] Wootton RJ. 1976. The biology of the sticklebacks. London: Academic Press.

[bib132] Wootton RJ. 1984. A functional biology of sticklebacks. Berkeley (CA): University of California Press.

[bib133] Wund MA , Valena S, Wood S, Baker JA. 2012. Ancestral plasticity and allometry in Threespine Stickleback reveal phenotypes associated with derived, freshwater ecotypes. Biol J Linn Soc. 105: 573–83. 10.5061/dryad.hb824gd4 PMC3351840 22611287

[bib134] Ziuganov V. 1991. The family *Gasterosteidae* of world fish fauna. Vol. 5 (1), Fauna of USSR. Fishes.

